# The relationship between wasting and stunting in young children: A systematic review

**DOI:** 10.1111/mcn.13246

**Published:** 2021-09-05

**Authors:** Susan Thurstans, Natalie Sessions, Carmel Dolan, Kate Sadler, Bernardette Cichon, Sheila Isanaka, Dominique Roberfroid, Heather Stobaugh, Patrick Webb, Tanya Khara

**Affiliations:** ^1^ Department of Population Health London School of Hygiene and Tropical Medicine London United Kingdom UK; ^2^ Emergency Nutrition Network Oxford UK; ^3^ Nutrition for Development UK; ^4^ No Wasted Lives/Action Against Hunger London UK; ^5^ Department of Epidemiology Harvard T.H. Chan School of Public Health Boston Massachusetts USA; ^6^ Department of Research Epicentre Paris France; ^7^ Faculty of Medicine University of Namur Namur Belgium; ^8^ Department of Food Technology, Safety and Health Ghent University Ghent Belgium; ^9^ Action Against Hunger USA New York New York USA; ^10^ Friedman School of Nutrition Science and Policy Tufts University Boston Massachusetts USA

**Keywords:** child growth, infectious disease, international child health nutrition, malnutrition, stunting, wasting

## Abstract

In 2014, the Emergency Nutrition Network published a report on the relationship between wasting and stunting. We aim to review evidence generated since that review to better understand the implications for improving child nutrition, health and survival. We conducted a systematic review following PRISMA guidelines, registered with PROSPERO. We identified search terms that describe wasting and stunting and the relationship between the two. We included studies related to children under five from low‐ and middle‐income countries that assessed both ponderal growth/wasting and linear growth/stunting and the association between the two. We included 45 studies. The review found the peak incidence of both wasting and stunting is between birth and 3 months. There is a strong association between the two conditions whereby episodes of wasting contribute to stunting and, to a lesser extent, stunting leads to wasting. Children with multiple anthropometric deficits, including concurrent stunting and wasting, have the highest risk of near‐term mortality when compared with children with any one deficit alone. Furthermore, evidence suggests that the use of mid‐upper‐arm circumference combined with weight‐for‐age *Z* score might effectively identify children at most risk of near‐term mortality. Wasting and stunting, driven by common factors, frequently occur in the same child, either simultaneously or at different moments through their life course. Evidence of a process of accumulation of nutritional deficits and increased risk of mortality over a child's life demonstrates the pressing need for integrated policy, financing and programmatic approaches to the prevention and treatment of child malnutrition.

**Key messages**

- A significant proportion of wasting and stunting is present at birth and can contribute to further growth failure during subsequent infancy and childhood. Improving maternal health and nutrition in pregnancy and early life could have a critical role in the prevention of wasting and stunting.
- Periods of wasting leave a child more likely to experience stunting and, to a lesser extent, vice versa. Common risk factors drive an accumulation of vulnerabilities. This underlines the need for cohesive policies and the implementation of services and activities to prevent both wasting and stunting.
- Concurrently wasted and stunted children have an elevated risk of death and should be considered as a high‐risk group in the targeting of treatment.
- A combination of weight‐for‐age *Z* score and mid‐upper‐arm circumference may be the most effective way to identify children at highest risk of mortality, including those concurrently wasted and stunted. Further evidence is needed to understand the operational implications.

## 1 INTRODUCTION

Undernutrition remains a major public health concern in many countries and an underlying cause of almost half of global child mortality (Black et al., 2013). The long‐term impacts of childhood undernutrition are far‐reaching, resulting in lower educational achievement, lower economic productivity and an increased risk of noncommunicable disease (Black et al., 2013; Murray et al., 2020; Victora et al., 2008) Current estimates suggest that 149 million children under 5 years are stunted and 49.5 million are wasted (Global Nutrition Report, 2020), while 15.9 million are experiencing both forms of undernutrition concurrently (Global Nutrition Report, 2018).

For many years, wasting and stunting have been viewed as separate conditions. As a result, the two have been largely disconnected within nutrition programmes, at policy and financing levels and in many areas of research without clear evidence supporting this distinction. The reasons for the historical shift from a previously more joined up way of looking at undernutrition (Waterlow, Gomez classification) are unclear, although some aspects of the divide have been entrenched by divergent funding and programmatic approaches in humanitarian and development contexts and the separation of wasting treatment and stunting prevention approaches (Wells, Briend, et al., 2019). In humanitarian contexts, the focus of programming tends to be on wasting treatment and mitigating acute mortality risk, whereas in stable development contexts, the bigger policy and programmatic focus is often on stunting prevention and the mitigation of associated longer‐term developmental deficits (SUN, 2016). That said, these divides are not typical of all settings and have started to lessen over recent years with growing attention to wasting treatment in developmental settings within health systems. There has also been more recent attention paid to wasting prevention and to issues of stunting in protracted crises with evidence highlighting that in fragile contexts, multiple forms of malnutrition coexist at high levels (Global Nutrition Report, 2020).

In 2014, the Emergency Nutrition Network (ENN) formed a technical interest group (TIG) of global experts (referred to as the WaSt TIG) to examine the relationship between wasting and stunting and published a technical briefing paper (Khara & Dolan, 2014) on the state of evidence, policy, research and programme implications of this relationship. This review concluded that wasting and stunting often coexist in the same child and that the risk of mortality associated with both wasting and stunting is heightened where they coexist. It also highlighted that there are common causal pathways, evidence pointing towards a direct relationship between wasting and stunting, that seasonality has a marked impact on both wasting and stunting prevalence and that in‐utero conditions and fetal growth contribute significantly to stunting and wasting at birth and during infancy. The report highlighted challenges around how wasting and stunting are commonly framed and reported, particularly the limitations of applying anthropometric cut‐offs at one point in time that fail to represent the process of wasting and/or stunting that a child may experience.

Since 2014, the WaSt TIG and other researchers have focused on more clearly defining the limitations posed by existing approaches to the framing of wasting and stunting. This process has raised critical research questions, including those around how children experience wasting and stunting throughout their life course and the implications of experiencing both. Our objective was to systematically review evidence generated since the original 2014 review to better understand the relationship between wasting and stunting in terms of both the physiological similarities and associations between the two as well as the implications of this relationship on interventions to improve child health, nutrition and survival. With such knowledge, mitigating different levels of risks through preventive approaches and treatment should be possible and, in so doing, would have global relevance towards the attainment of World Health Assembly (WHA) and Sustainable Development Goals (SDG) targets as they relate to wasting and stunting.

## 2 MATERIALS AND METHODS

We conducted a systematic review following the *Preferred Reporting Items for Systematic reviews and Meta‐Analyses* (PRISMA) guidelines (Moher et al., 2009). A review protocol was developed, in coordination with a subworking group (SWG) of experts from the WaSt TIG to define the scope of the review. The protocol was registered with the PROSPERO International prospective register of systematic reviews (CRD42019153330).

### 2.1 Search strategy

**Figure 1:**
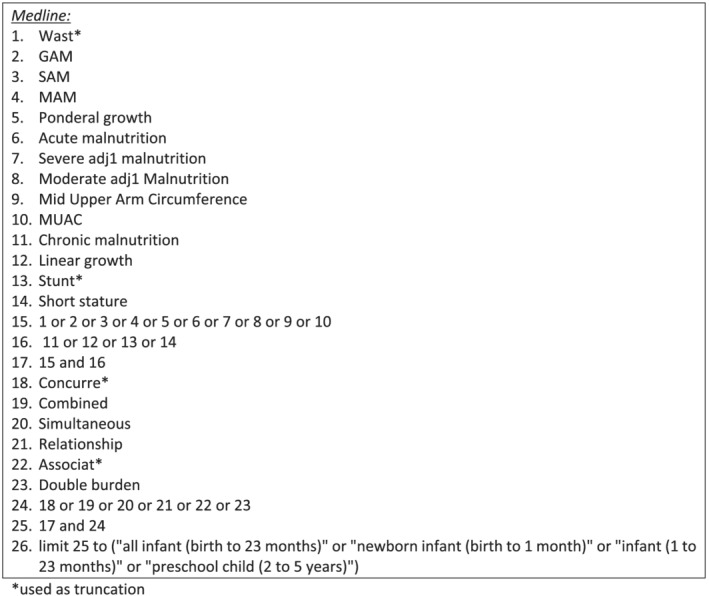
Search strategy

We identified search terms to describe wasting and stunting and the relationship between the two conditions, including implications for ponderal and linear growth. The search terms are listed in Figure 1. We searched Medline, Embase and global health databases through Ovid, applying limits for studies published after 2012 to allow for any studies that may have been missed in the 2014 review and for the age of the individuals included in studies. We also issued a call for studies known to the WaSt TIG members in May 2020. The final search was conducted in June 2020. Both the search strategy and the eligibility criteria were guided by the Population, Intervention, Comparison, Outcome (PICO) framework in order to delineate the question of focus for the review and to define inclusion and exclusion criteria. The PICO is presented in Table 1.

**Table 1:** Population, Intervention, Comparison, Outcome

Population	Children 0–5 years Low‐ and middle‐income countries
Intervention	Assessment, review or treatment of wasting, stunting, concurrent wasting and stunting
Comparison	No comparison
Outcome	Incidence, prevalence, treatment outcome measures (recovery, mortality, length of stay etc.), morbidity, concurrent wasting and stunting

### 2.2 Eligibility criteria

We reviewed studies from low‐ and middle‐income countries (LMICs) where wasting and stunting are most prevalent. As wasting and stunting commonly occur in children under 5 years of age, we applied age limits from 0 to 59 months. We considered studies in the review that assessed both ponderal growth/wasting and linear growth/stunting as well as the association between the two. Included studies focused on prevalence, physiological mechanisms and outcomes related to growth and mortality. We included all types of studies that involved primary research (case control studies, cross‐sectional studies and secondary data analyses). We also included reviews if they presented pooled analysis or new insights into the relationship between wasting and stunting. Both peer‐reviewed papers and grey literature identified through the search were considered for inclusion.

We excluded studies that assessed wasting and stunting separately and that did not report on either condition in relation to the other. Also excluded were studies that focused on obesity, micronutrients, those that included children over 5 years of age and published abstracts and viewpoints.

In most of the literature reviewed, wasting is defined as low weight‐for‐length/height (WLZ/WHZ) (<−2 *Z* score of the reference), stunting as low height‐for‐age (HAZ) (<−2 *Z* score of the reference) and concurrent wasting and stunting as both low WHZ and HAZ at the same time (<−2 *Z* score WHZ and <−2 *Z* score HAZ). However, in literature referring to treatment programmes targeting wasting, standard WHO mid‐upper‐arm circumference (MUAC) criteria for defining wasting may also be included. We have endeavoured to specify literature for which the latter is the case.

### 2.3 Study selection, data extraction and analysis

All records identified during the search were exported into EndNote (EndNote V.X8, Clarivate Analytics) and duplicates removed. Initial screening of titles and abstracts was conducted by ST to identify studies unrelated to the scope of the review. The remaining studies were then independently screened by ST and NS by reading the full texts. Discrepancies were resolved via discussion and, where necessary, a third reviewer (TK) was consulted. A data extraction template was developed in Excel, piloted and reviewed by members of the research team before full extraction took place.

We identified three main themes before extraction: physiological understanding of the similarities in wasting and stunting, the interrelationship between the two conditions and the implications of this relationship and then extracted data along these lines. We extracted data into an Excel spreadsheet including information on authors, titles, dates of publication, sample size, data/information relevant to each theme and any research recommendations and conclusions.

### 2.4 Risk of bias assessment

We assessed the quality of included studies using an adapted version of the SIGN checklists (https://www.sign.ac.uk/what-we-do/methodology/checklists/). We selected the SIGN checklists as they provide a checklist of items for case–control and cohort studies, study designs commonly used in the studies selected for this review. Adaptation was necessary due to the varied nature of the studies included. We assessed factors such as clearly defined objectives, study design, definition of participants, exposures and outcomes, statistical methods, addressing of bias and potential confounders and the presentation of results.

## 3 RESULTS

### 3.1 Study selection

**Figure 2:**
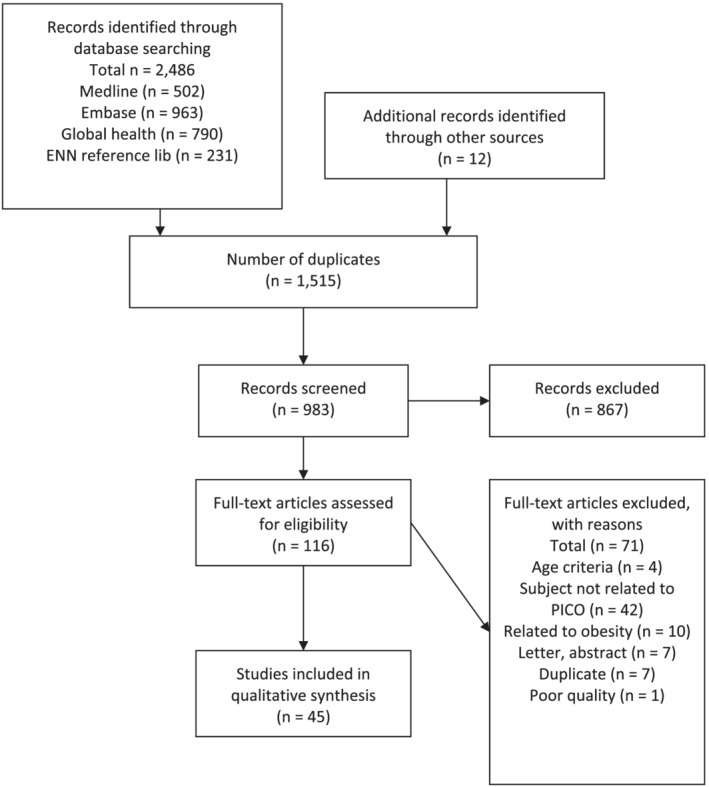
PRISMA flow diagram

The results of the search process are presented in Figure 2. The database search identified 2486 studies and reports and an additional 12 studies came from WaSt TIG members. After removing duplicates, 983 studies and reports remained, of which 867 were excluded following initial screening. One hundred and sixteen full text studies and reports were assessed for eligibility of which 71 were excluded. The reasons for exclusion are given in Figure 2. We included a total of 45 studies and reports in our final review.

### 3.2 Study characteristics and risk of bias

We present the characteristics of each included study or report in Table 2. We included a total of 39 peer reviewed studies, one manual chapter, three preprint publications and two published reports (both appearing in ENN's Field Exchange ‘peer‐to‐peer’ publication, https://www.ennonline.net/fex). These included 21 cross sectional and 18 longitudinal studies. In total, 14 countries were covered in studies and reports conducted in single countries while 18 studies covered multiple countries—the largest analysis covering 84 countries (Khara et al., 2018). The risk of bias assessment is presented in Table 3. Overall, we assessed the studies and reports selected to be of ‘acceptable’ quality according to the adapted SIGN criteria, but one study was excluded on the basis of quality (Carroll et al., 2012).

**Table 2:** Study characteristics

No	1st author and year	Study design	Sample size	Country	Age range of sample	Physiology	Relationship	Mortality	Burden	Age and sex	Anthropometric indices	Treatment outcomes	Fat accumulation	Research gaps	Key findings	Key conclusions
1	Angood (2016)	CHNRI	NA	NA	NA									✓	Priority research questions for wasting and stunting identified	Research is needed into wasting and stunting in order to inform global health efforts to address undernutrition
2	Benjamin‐Chung et al. (2020)	Longitudinal	62,993	Multiple	0–24 months			✓	✓	✓					The highest incidence of stunting onset occurred from birth to 3 months of age. From 0 to 15 months of age, less than 5% of children per month reversed their stunting status and, among those who did, stunting relapse was common.	Preventive intervention within prenatal and early postnatal phases, coupled with continued delivery of postnatal interventions through the first 1000 days of life, are key to overcoming the early occurrence and low reversal rates of stunting.
3	Binns and Myatt (2018)	Longitudinal	163	Malawi	6–59 months								✓		No incidence of overweight or adiposity.	Children age ≥6 months with a height less than 65 cm will not become overweight or obese following RUTF treatment with MUAC as admission and discharge criteria.
4	Briend (2012)	Review	NA	NA		✓								✓	Fat loss and muscle mass loss are associated with both wasting and stunting. Hormones produced by fat play a crucial role in immune function and bone growth which might explain reduced linear growth in the case of low WHZ.	Crucial to prevent both wasting and stunting in order to reduce malnutrition related mortality.
5	Christian et al. (2013)	Cohort	58,317	Multiple	0–59 months			✓							LBW was associated with higher odds of wasting, stunting and underweight.	Childhood undernutrition may have its origins in the fetal period, indicating the need for early intervention and targeting of adolescent girls and pregnant women with interventions known to reduce FGR and preterm birth.
6	Fabiansen et al. (2017)	RCT	1609	Burkina Faso	6–23 months								✓		Compared CSB and LNS and assessed body composition to determine the quality of weight gain with fat free mass tissue accretion as a primary outcome. The findings showed that fat free tissue accretion as well as recovery from MAM were both higher using LNS compared with CSB.	Findings support wider use of LNS in MAM treatment.
7	Fabiansen (2020)	Cohort	1609	Burkina Faso	6–23 months								✓		No difference found in fat accumulation between tall and short children when treated using RUTF.	Short children do not gain excessive fat during supplementation. The use of length as eligibility criteria for treatment should be discontinued and all children ≥6 months with low MUAC should be included in SFPs.
8	Garenne (2020)	Cross‐sectional	37,670	Senegal	12–59 months		✓	✓	✓	✓					Children in Senegal are taller but thinner. Changes in weight and height were most apparent in poorer households. Findings were consistent with reduction in mortality.	Control of stunting likely a result of control of infectious disease. Increase in reduced WHZ requires further investigation.
9	Garenne (2018)	Longitudinal	12,638	Senegal	6–59 months	✓	✓	✓	✓	✓	✓			✓	Wasting and stunting are correlated. Concurrent wasting and stunting peaks around 30 months and is higher in boys than girls, but this difference could not be explained by muscle mass or fat mass measured by arm or muscle circumference, triceps or subscalpular skinfold.	Concurrent wasting and stunting is a strong risk factor for mortality.
10	Harding et al. (2018a)	Cross‐sectional	62,509	Multiple: South Asia	0–59 months				✓						Key determinants of child stunting are also significant determinants of child wasting in Asia.	The co‐occurrence of wasting and stunting requires more integrated interventions. That is, programmes aimed at preventing LBW and poor IYCF to avert stunting should be linked more effectively with actions aimed at the management of wasting.
11	Harding et al. (2018b)	Cross‐sectional	252,797	Multiple: South Asia	0–59 months				✓						LBW strongly associated with wasting and wasting and stunting.	Programmes aimed at preventing LBW and poor IYCF (to reduce stunting) should be linked with actions aimed at the management of wasting.
12	Imam et al. (2020)	Cross‐sectional	472	Nigeria	6–59 months				✓	✓					Stunting prevalence is high among severely wasted children attending CMAM programmes in North‐Western Nigeria.	CMAM programmes should adapt to consider stunting as well as wasting.
13	Isanaka et al. (2019)	Cohort	1542	Niger	6–59 months		✓		✓			✓			High burden of stunting in wasting treatment programme. Stunting did not impair response to treatment. There was limited linear growth in this population.	There is a direct relationship whereby inadequate weight is associated with slowed linear growth. Wasting contributes to stunting.
14	Kangas et al. (2020)	RCT	802	Burkina Faso	6–59 months								✓		Half of weight gained by children during SAM treatment was fat free mass (FFM) and the FFM of treated children at recovery was similar to community controls indicating incomplete FFM recovery during SAM treatment.	There is no evidence from this study of a differential effect of a reduced RUTF dose on the tissue accretion of treated children when compared with standard treatment suggesting that, in a relatively food secure context, a reduction in the RUTF dose can result in similar body composition by recovery.
15	Kassie and Workie (2019)	Cross‐sectional	8768	Ethiopia	0–59 months		✓								Underweight was associated with both stunting and wasting. There was no association between stunting and wasting. There is no a three way interaction among stunting, wasting and underweight.	Wasting, stunting and underweight should be considered simultaneously to estimate the actual burden of childhood undernutrition.
16	Khara (2017)	Cross‐sectional	198,005,973	Multiple	6–59 months				✓	✓					Concurrent wasting and stunting highest in 12–24 months age group and males. Fragile and conflict affected states have higher concurrence than stable countries.	Concurrent wasting and stunting represents a high risk group. Investigations needed to ensure this group is being reached.
17	Kinyoki (2016)	Cross‐sectional	73,778	Somalia	6–59 months				✓						Determinants of wasting are similar but patterns in correlation are variable.	Wasting, stunting and underweight have common risk factors. Joined up programming is required to address wasting and stunting.
18	Kosek and Mal‐Ed Network Investigators (2017)	Cohort	1253	Multiple	0–24 months	✓									Higher burdens of enteropathogens were associated with elevated biomarker concentrations of gut and systemic inflammation and indirectly associated with both reduced linear and ponderal growth.	The strongest evidence for environmental enteropathy was the association between enteropathogens and linear growth mediated through systemic inflammation.
19	Lelijveld et al. (2016)	Cohort	320	Malawi			✓					✓			More stunting found in case group. Sitting height was similar across groups suggesting preservation of torso growth.	SAM has long term adverse effects on growth and body composition.
20	McDonald et al. (2013)	Meta‐analysis of cohort studies	53,767	Multiple	0–59 months			✓	✓						Hazarad ratio for stunting, wasting and underweight was 12.3.	Children with multiple anthropometric deficits are at increased risk of mortality.
21	Mertens, Benjamin‐Chung, Colford, Coyle, et al., (2020)	Longitudinal	108,336	Multiple	0–24 months			✓	✓	✓					Children who experienced early ponderal or linear growth failure were at higher risk of persistent growth failure and were more likely to die by age 24 months.	A focus on pre‐conception and pregnancy is key for preventive interventions.
22	Mertens, Benjamin‐Chung, Colford, Hubbard, et al., (2020)	Longitudinal	10,854	Multiple	0–24 months		✓		✓	✓		✓			Wasting incidence is five‐fold higher than prevalence estimates suggest. Peak incidence is between 0 and 3 months.	New focus is required to extend preventive interventions for child wasting to pregnant and lactating mothers and children below age 6 months.
23	Mutunga (2020)	Cross‐sectional	47,481	Multiple	0–59 months				✓						Concurrent wasting and stunting is prevalent in Southeast Asia.	Both preventive and curative approaches are needed to address wasting in Southeast Asia.
24	Myatt et al. (2019)	Longitudinal	5751	Senegal	0–59 months			✓			✓				MUAC and WAZ detected all near‐term deaths associated with anthropometric deficits, including concurrent wasting and stunting.	Therapeutic feeding programmes should consider WAZ and MUAC admission criteria.
25	Myatt et al. (2018)	Cross‐sectional	1,796,991	Multiple	6–59 months		✓	✓		✓	✓				Children who are wasted and stunted are also underweight. Concurrently wasted and stunted children have a high risk of mortality.	Therapeutic feeding programmes should include concurrent wasting and stunting given the high risk of mortality.
26	Nandy and Svedberg (2012)	Cross‐sectional	45,377	India	0–59 months	✓									The CIAF supports the assessment of the relationship between malnutrition, morbidity and poverty.	Efforts to reduce poverty and increase living standards are needed to support reduction of malnutrition.
27	Ngari et al. (2019)	Longitudinal	1169	Kenya	2–59 months		✓					✓			No significant increase in HAZ at 1 year follow‐up after inpatient treatment for complicated SAM, despite MUAC growth and weight gain. Linear growth was associated with less severe wasting and more stunted and with fewer comorbidities at admission.	Intensive nutritional rehabilitation did not resolve stunting.
28	Ngwira et al. (2017)	Cross‐sectional	4861	Malawi	0–59 months		✓								Associations found between wasting and underweight and stunting and underweight but no association found between wasting and stunting.	Wasting, stunting and underweight are valid measures which cannot represent each other.
29	O'Brien et al. (2020)	Longitudinal	1023	Niger	6–60 months			✓			✓				MUAC was the strongest predictor of mortality followed by WAZ.	MUAC is a better predictor of mortality in this study population.
30	Odei Obeng‐Amoako, Karamagi, et al. (2020)	Cross‐sectional	32,962	Uganda	6–59 months		✓		✓	✓	✓				All concurrent wasted and stunted children were also underweight. Concurrent wasting and stunting prevalence of 5% raises public health concerns. WaSt was more common among younger children and males, but the majority of WaSt children with low MUAC were female.	Consider the integration of WAZ into therapeutic feeding programmes to detect and treat concurrent wasting and stunting.
31	Odei Obeng‐Amoako, Myatt, et al. (2020)	Cohort	788	Uganda			✓	✓	✓	✓	✓	✓			High number of stunted children in wasting treatment programme.	Existing therapeutic feeding protocols can be used to detect and effectively treat children with concurrent wasting and stunting.
32	Odei Obeng‐Amoako, Wamani, et al. (2020)	Cross sectional	33,054	Uganda	6–59 months		✓		✓	✓					Factors associated with concurrent wasting and stunting included male sex, age between 12 and 59 months, acute respiratory infection, diarrhoea, malaria/fever, maternal underweight, maternal short stature, low MUAC (<23 cm) and mother having ≥4 live‐births.	Preventing concurrent wasting and stunting through pragmatic and joint approaches is critical. Future prospective studies should focus on risk factors in order to inform effective prevention strategies
33	Pomati and Nandy (2019)	Cross‐sectional	28 DHS samples	Multiple	0–59 months			✓	✓		✓				The mortality risk attached to multiple anthropometric deficits is high including children who are wasted and underweight and stunted and underweight.	CIAF identifies children under five with a higher risk of mortality.
34	Prentice et al. (2013)	Cross‐sectional	227	Multiple	0–24 months	✓	✓							✓	Infants born with growth deficits will likely continue to have growth deficits as they progress along growth trajectories.	Research is needed to understand causal pathways to growth faltering.
35	Reese‐Masterson et al. (2016)	Cross‐sectional	227	Kenya	0–24 months				✓						A small sub‐sample of the population was found to be both wasted and stunted.	The study makes recommendations for programme‐specific data and measurement‐related improvements to enable more meaningful analysis.
36	Richard, Black, & Checkley (2012)	Longitudinal	1599	Multiple	0–24 months		✓								Children with wasting only in early life had similar LAZ at 18–24 months than those with no wasting. More recent wasting was associated with lower LAZ.	Wasting is associated with the process of stunting. Prevention of wasting could increase attained stature in children.
37	Roberfroid (2015)	Cross‐sectional	14,409	Multiple	6–59 months					✓	✓				MUAC <125 mm should not be used as a stand‐alone criteria for wasting given its strong association with age, sex and stunting and its low sensitivity to detect slim children.	Further research is needed to better understand the clinical and physiological outcomes of the various anthropometric indicators of malnutrition.
38	Saaka and Galaa (2016)	Cross‐sectional	2720	Ghana	0–59 months		✓		✓						Children who were wasted were more at risk of stunting.	WHZ relates to linear growth. Stunting and wasting share common determinants therefore prevention of both wasting and stunting will positively influence linear growth.
39	Sage (2017)	Cross‐sectional	6602	Guinea‐Bissau	6–59 months				✓	✓					Associations that were insignificant for wasting and stunting individually were significant for concurrent wasting and stunting. Mosquito nets and lack of diarrhoea in the last two weeks were both protective of concurrent wasting and stunting.	Concurrent wasting and stunting should be a key consideration for nutrition programming in Guinea‐Bissau.
40	Schoenbuchner et al. (2019))	Longitudinal	5160	Gambia	0–24 months		✓		✓	✓	✓			✓	Being wasted was predictive of stunting, even accounting for current stunting. Boys more likely to be wasted, stunted and underweight than girls, and are more susceptible to seasonally driven growth deficits.	Stunting is in part a biological response to previous wasting highlighting the policy implications of recognising the importance of wasting.
41	Schwinger (2019)	Longitudinal	15,060	Multiple	6–59 months			✓							Children who have a low WHZ but a MUAC above the cut‐off would be omitted from diagnosis and treatment.	In addition to simple tools for case finding, the use of WHZ should be used whenever possible.
42	Shively (2017)	Cross‐sectional	11,946	Uganda and Nepal	0–59 months		✓								Nutritional status was sensitive to rainfall, more so in Uganda than Nepal.	Further research is needed to understand the heterogeneity in results and the role of economic development in promoting child nutrition.
43	Stobaugh et al. (2018)	Longitudinal	1487	Malawi	6–62 months		✓					✓			Children with poor linear growth after MAM are more likely to experience relapse.	Wasting contributes to stunting.
44	Victora (2015)	Cross‐sectional	24,817 girls and 26,378 boys	Multiple	Newborns				✓						Stunting and wasting are separate anthropometric phenotypes with intrauterine origins. Larger studies in higher risk populations may strengthen the associations between wasting and stunting and will also reinforce the differences.	Newborns should be classified using both wasting and stunting measures.
45	Wells (2019)	Review	NA	NA	NA	✓	✓						✓		There are different potential pathways which underlie the association with stunting and future body composition including environmental drivers, changes in growth and tissue masses or alterations in metabolic pathways.	Further research is needed in relation to the functional significance of FFM and fat mass for survival, physical capacity and noncommunicable disease risk.

**Table 3:** Risk of bias assessment

Study	The study addresses an appropriate and clearly focused question	Is the study design clearly described?	Where relevant, are groups being studied selected from source populations that are comparable in all respects other than the factor under investigation?	Are eligibility criteria for participants well described (including controls where relevant)?	Are outputs, exposures and potential confounders well described?	Are sources of data and methods of assessment or measurement clearly described?	Are efforts to address potential bias or confounding described?	Is study size clearly stated and an explanation given for study size?	Is a clear description of statistical methods provided including, where appropriate, how missing data and subgroups were handled and how match of cases and controls was addressed and any sensitivity analysis?	Were the number of participants at each stage of the study (including loss to follow‐up) well described?	Were characteristics of the study participants described?	Were outcome indicators clearly reported?	Have estimates (adjusted where relevant) and associated confidence intervals been reported?	Were study limitations recognised?
Angood (2016)	●	●	NA	NA	NA	●	NA	NA	NA	NA	NA	●	NA	●
Benjamin‐Chung (2020)	●	●	●	●	●	●	●	●	●	●	●	●	●	●
Binns and Myatt (2018)	●	●	●	●	●	●	●	●	●	●	●	●	●	●
Briend (2012)	●	NA	NA	NA	NA	NA	NA	NA	NA	NA	NA	NA	NA	NA
Christian et al. (2013)	●	●	●	●	●	●	●	●	●	●	●	●	●	●
Fabiansen et al. (2017)	●	●	●	●	●	●	●	●	●	●	●	●	●	●
Fabiansen (2020)	●	●	●	●	●	●	●	●	●	●	●	●	●	●
Garenne (2020)	●	●	●	●	●	●	●	●	●	●	●	●	●	●
Garenne (2018)	●	●	●	●	●	●	●	●	●	●	●	●	●	●
Harding et al. (2018a)	●	●	●	●	●	●	●	●	●	●	●	●	●	●
Harding et al. (2018b)		●	●	●	●	●	●	●	●	●	●	●	●	●
Imam et al. (2020)	●	●	●	●	●	●	●	●	●	●	●	●	●	●
Isanaka et al. (2019)	●	●	●	●	●	●	●	●	●	●	●	●	●	●
Kangas et al. (2020)	●	●	●	●	●	●	●	●	●	●	●	●	●	●
Kassie and Workie (2019)	●	●	●	●	●	●	●	●	●	●	●	●	●	●
Khara (2017)	●	●	●	●	●	●	●	●	●	●	●	●	●	●
Kinyoki (2016)	●	●	●	●	●	●	●	●	●	●	●	●	●	●
Kosek and Mal‐Ed Network Investigators (2017)	●	●	●	●	●	●	●	●	●	●	●	●	●	●
Lelijveld et al. (2016)	●	●	●	●	●	●	●	●	●	●	●	●	●	●
McDonald et al. (2013)	●	●	●	●	●	●	●	●	●	●	●	●	●	●
Mertens, Benjamin‐Chung, Colford, Coyle, et al., (2020)	●	●	●	●	●	●	●	●	●	●	●	●	●	●
Mertens, Benjamin‐Chung, Colford, Hubbard, et al., (2020)	●	●	●	●	●	●	●	●	●	●	●	●	●	●
Mutunga (2020)	●	●	●	●	●	●	●	●	●	●	●	●	●	●
Myatt et al. (2019)	●	●	●	●	●	●	●	●	●	●	●	●	●	●
Myatt et al. (2018)	●	●	●	●	●	●	●	●	●	●	●	●	●	●
Nandy and Svedberg (2012)	●	●	NA	NA	NA	NA	NA	NA	NA	NA	NA	NA	NA	NA
Ngari et al. (2019)	●	●	●	●	●	●	●	●	●	●	●	●	●	●
Ngwira et al. (2017)	●	●	●	●	●	●	●	●	●	●	●	●	●	○
O'Brien et al. (2020)	●	●	●	●	●	●	●	●	●	●	●	●	●	●
Odei Obeng‐Amoako, Karamagi, et al. (2020a)	●	●	●	●	●	●	●	●	●	●	●	●	●	●
Odei Obeng‐Amoako, Myatt, et al. (2020)	●	●	●	●	●	●	●	●	●	●	●	●	●	●
Odei Obeng‐Amoako, Wamani, et al. (2020)	●	●	●	●	●	●	●	●	●	●	●	●	●	●
Pomati and Nandy (2019)	●	●	●	●	●	●	●	●	●	●	●	●	●	●
Prentice et al. (2013)	●	◐	NA	NA	NA	NA	NA	NA	NA	NA	NA	NA	NA	NA
Reese‐Masterson et al. (2016)	●	●	●	●	●	●	○	●	●	●	●	●	●	●
Richard, Black, & Checkley (2012)	NA	NA	NA	NA	NA	NA	NA	NA	NA	NA	NA	NA	NA	NA
Roberfroid (2015)	●	●	●	●	●	●	●	●	●	●	●	●	●	●
Saaka and Galaa (2016)	●	●	●	●	●	●	●	●	●	●	●	●	●	●
Sage (2017)	●	●	●	●	●	●	●	●	●	●	●	●	NA	●
Schoenbuchner et al. (2019)	●	●	●	●	●	●	●	●	●	●	●	●	●	○
Schwinger (2019)	●	●	●	●	●	●	●	●	●	●	●	●	●	●
Shively (2017)	●	●	●	●	●	●	●	●	●	●	●	●	●	○
Steenkamp, 2016	●	●	◐	◐	○	◐	○	◐	◐	○	◐	●	●	●
Stobaugh et al. (2018)	●	●	●	●	●	●	●	●	●	●	●	●	●	●
Victora (2015)	●	●	●	●	●	●	●	●	●	●	●	●	●	●
Wells (2019)	●	●	NA	NA	NA	NA	NA	NA	NA	NA	NA	NA	NA	NA

*Note*: ● = Yes, ◐ = partially, ○ = no.

### 3.3 Interconnected physiological processes

We reviewed studies that considered the physiological processes underlying the potential interaction between wasting and stunting, either as the primary objective or within the discussion. While little was identified in the way of epidemiological research in this area, published narrative reviews provided some discussion of the possible physiological mechanisms.

Infectious disease has long been recognised as both a cause and consequence of undernutrition. Infectious disease can result in both wasting and stunting through decreased or altered nutritional intake, impaired intestinal absorption and increased metabolism from fever, immune response and environmental enteropathy (Kosek and Mal‐Ed Network Investigators, 2017; Nandy & Svedberg, 2012) resulting in a higher risk of mortality (Harding et al., 2018b). Conversely, undernourished children are more vulnerable to infectious disease due to the impairment of their immune system. Children identified as concurrently wasted and stunted have been found to be at increased risk of infectious disease (Harding et al., 2018b; Odei Obeng‐Amoako, Karamagi, et al., 2020; Sage, 2017).

The literature describes the association between loss of fat mass and wasting and stunting, although inconsistently so in the case of stunting (Briend, Khara et al., 2015; Fabiansen et al., 2016, 2017; Wells, 2019). Fat plays a role in the maintenance of the immune system, which demands increased energy when stimulated by infection. This suggests that fat depletion acts as an additional mechanism linking wasting and stunting with increased infection and mortality (Briend, Khara et al., 2015). Muscle mass loss also occurs in both wasting and stunting, particularly in the case of infection when protein breakdown is increased due to an increased need for amino acids to build the proteins involved in the immune response. As muscle mass is positively associated with age, infants have low levels and are therefore especially vulnerable to the effects of undernutrition and associated mortality (Briend, Khara et al., 2015).

The literature also describes the role of leptin in the relationship between wasting and stunting linked to the above body composition changes above. Leptin is a hormone produced primarily by fat cells and is responsible for the regulation of energy, hunger and metabolism as well as playing a central role in stimulating immune function and linear growth (Wells, Briend, et al., 2019). The levels of leptin produced reflect body fat stores and indeed low levels of leptin are noted alongside the deficits in fat and muscle mass in wasted and stunted children. Furthermore, low levels of leptin in children with undernutrition are predictive of an increased risk of mortality (Briend, Khara et al., 2015) and implicated in slowed linear growth during wasting (Wells, 2019).

Briend, Akomo, et al. (2015) also highlight the coexistence of stunting and high overweight prevalence. They suggest that high fat stores alone are insufficient to support linear growth and that low intake of nutrients such as zinc, sulphur, phosphorous, vitamins D, C and K and copper—nutrients needed for bone growth and lean tissue synthesis—may explain the association between stunting with reduced muscle mass and normal or increased fat reserves. Leptin might also have an effect on bone growth which might explain the reduced linear growth observed in wasted children and the frequent association with stunting (Briend, Khara et al., 2015).

### 3.4 The burden, aetiology and timing of wasting and stunting

Cross‐sectional population‐based surveys are often used to determine prevalence and associated risk factors for wasting and stunting despite being known to be potentially problematic in underestimating the true burden of acute conditions like wasting (Action Against Hunger, 2018; Khara et al., 2018). A recent pooled longitudinal analysis (Mertens, Benjamin‐Chung, Colford, Hubbard, et al., 2020) highlights the challenge in the interpretation of cross‐sectional data for wasting and demonstrates that these methods fail to measure the onset, recovery and persistence (defined as persistent if ≥50% of WLZ measurements from birth to 24 months fell below −2) of wasting. Data showed that the cumulative incidence of wasting in children under 24 months was 33%, more than five times higher than prevalence which was 6%. This means that the burden of wasting is likely far higher than traditional cross‐sectional studies suggest. In the case of stunting, the data suggest that prevalence estimates matched general patterns of cross‐sectional studies, gradually increasing with age and therefore are a more accurate estimate of true burden (Benjamin‐Chung et al., 2020).

The same analyses provide evidence that challenges a reliance on prevalence estimates to inform interventions. It is often understood that wasting peaks at 12–23 months (Garenne et al., 2019; Schoenbuchner et al., 2019). However, by looking at the incidence of wasting, the study established that peak incidence is between birth and 3 months (Mertens, Benjamin‐Chung, Colford, Hubbard, et al., 2020). A similar analysis of stunting (Benjamin‐Chung et al., 2020) also offers new insights into the timing of linear growth faltering, typically understood to be highest between 6 and 24 months. Data showed that the incidence of stunting was also highest from birth to 3 months. Although some children went on to experience stunting reversal, they later continued to experience linear growth faltering and more than 20% were stunted again at later measurements. These results emphasise the need for preventive and therapeutic interventions that usually target children from 6 to 59 months to include children under 6 months while also extending inclusion of both prevention and treatment of undernutrition in women of reproductive age, as well as pregnant and lactating women.

As with the 2014 review, studies in this review assessing the aetiology of wasting and stunting and concurrent wasting and stunting demonstrate that many of the driving factors are common (Harding et al., 2018b; Mertens et al., 2020; Saaka & Galaa, 2016; Shively, 2017). Underlying factors such as poor maternal nutrition (Mertens, Benjamin‐Chung, Colford, Coyle, et al., 2020), high parity (Mertens et al., 2020), low education levels (Mertens, Benjamin‐Chung, Colford, Coyle, et al., 2020; Odei Obeng‐Amoako, Karamagi, et al., 2020), low birth weight (LBW) and/or length (Mertens, Benjamin‐Chung, Colford, Coyle, et al., 2020) and poor feeding practices (Harding et al., 2018a; Prentice et al., 2013) (Saaka & Galaa, 2016) have all been shown to be associated with both wasting and stunting and concurrent wasting and stunting in cross‐sectional analysis. Likewise, poor socio‐economic conditions (Mertens, Benjamin‐Chung, Colford, Coyle, et al., 2020) and seasonality (Schoenbuchner et al., 2019) contribute to both conditions.

### 3.5 Evidence for the relationship between wasting and stunting

Population‐level analyses of the association between wasting and stunting have in the past led to conclusions that the two conditions were unrelated, largely due to the perceived separation in prevalence and distribution patterns across populations. Cross‐sectional results have been inconsistent in demonstrating any association between the two, with some single country studies showing low or no association between wasting and stunting (Kassie & Workie, 2019; Ngwira et al., 2017; Reese‐Masterson et al., 2016). Population‐level datasets mined specifically to explore the pertinent relationships now support a link between wasting and stunting that is more than just chance or random statistical noise. A large cross‐sectional study involved analysis of 51 countries and shows the existence of a relationship whereby wasting and stunting were positively and significantly associated with each other in 37 of 51 countries (Myatt et al., 2018). Longitudinal studies in this review are also supportive of a relationship between the two conditions. In Senegal, a two‐way dose response relationship was found whereby the proportion of wasted children increases with the degree of stunting and the proportion of stunting increases with the degree of wasting (Garenne et al., 2019). Within treatment programmes for severe acute malnutrition (SAM), evidence of a relationship is also apparent. Several analyses of children admitted into outpatient and/or inpatient feeding programmes indicate children with SAM are often stunted (Isanaka et al., 2019; Ngari et al., 2019; Schoenbuchner et al., 2019) (see Table 2). In Malawi, a strong association was found between poor linear growth and relapse to SAM and to moderate acute malnutrition (MAM) although the exact direction of this relationship was unclear (Stobaugh et al., 2018).

### 3.6 Wasting leading to stunting

We identified a number of studies that are supportive of a direct relationship between wasting and stunting whereby episodes of wasting contribute to stunting including one review (Richard, Black, & Checkley, 2012), one cross‐ sectional (Saaka & Galaa, 2016) and six longitudinal studies (Isanaka et al., 2019; Ngari et al., 2019; Schoenbuchner et al., 2019; Richard, Black, Gilman, et al., 2012; Mertens et al., 2020; Mertens, Benjamin‐Chung, Colford, Coyle, et al., 2020). Longitudinal data from The Gambia showed that being wasted was predictive of stunting within the next three‐month period by a factor of 3.2 after accounting for current stunting status (Schoenbuchner et al., 2019). Multicountry longitudinal analysis showed that persistent wasting from birth to 6 months (defined as >50% of measurements wasted) was strongly associated with incident stunting at older ages (Mertens, Benjamin‐Chung, Colford, Coyle, et al., 2020). Both studies indicate a time lagged effect whereby wasting is followed by stunting.

One hypothesis from these studies suggests that the body's response to weight faltering is to slow or halt linear growth until weight is gained and any infection is treated. In other words, weight (lean and fat mass) can be regained or maintained during nutritional stress at the expense of linear (height/length) growth (Isanaka et al., 2019; Richard, Black, & Checkley, 2012). Analysis from Niger tracking linear growth during wasting treatment suggests that HAZ deteriorates during the period of rapid weight gain that accompanies rehabilitation (Isanaka et al., 2019). Linear growth that was observed during periods of SAM treatment was characterised by children who were less wasted and less stunted (Isanaka et al., 2019; Ngari et al., 2019) and had fewer comorbidities at baseline (Ngari et al., 2019), suggesting that on top of the level of wasting and prior stunting, untreated comorbidities may also hold back linear growth. Population‐level data from Senegal supports this suggestion showing trends in linear growth that increased with improving health status (Garenne, 2020).

These studies and data from The Gambia (Schoenbuchner et al., 2019) also suggest that the effect of episodes of wasting on linear growth is modified by age, where wasting appears to be more detrimental to long‐term linear growth the later it happens, and recovery of HAZ is more likely if wasting occurs early and not subsequently. For example, a longitudinal study of infants and children 0–24 months in LMIC countries (Richard, Black, Gilman, et al., 2012) found no long‐term effect of one period of wasting in the first 6 months of life on length‐for‐age *Z* score (LAZ) at 18–24 months if no further wasting was experienced after that time, suggesting that one episode of wasting in this age group is not enough to slow/halt linear growth. However, wasting after 6 months of age, and greater variability in WLZ in the first 17 months of life, was associated with lower LAZ at 18–24 months. Seasonal evidence also suggests that wasting is associated with further wasting whereby infants who were wasted in the first wet season of their life were more likely to be wasted in their second wet season, even after controlling for whether they were wasted during the intervening dry season (Schoenbuchner et al., 2019).

### 3.7 Stunting leading to wasting

We also identified evidence to support a direct relationship whereby stunting leads to wasting, although the physiological mechanisms are less clear for this direction of the relationship. We identified two longitudinal studies demonstrating a strong and significant effect of stunting on the risk of subsequent wasting (Garenne et al., 2019; Schoenbuchner et al., 2019). The degree of stunting affected the level of risk with more severe stunting more likely to result in wasting.

### 3.8 Concurrent wasting and stunting

Some studies conducted since the 2014 review have focused on identifying the burden and implications of concurrent wasting and stunting. We identified studies that explored the prevalence and distribution of concurrent wasting and stunting at population level or within SAM treatment programmes (results are presented in Table 4). These studies cover a wide geographical area with 17 covering multiple countries. The largest population prevalence study includes analysis of 84 countries and indicates that fragile and conflict affected states (FCAS) appear to be disproportionately affected with higher rates of concurrent wasting and stunting than stable contexts (pooled prevalence 3.6%, 95% CI [3.5, 3.6] in FCAS compared with 2.24%, 95% CI [2.18, 2.30] in stable contexts *p* value <0.0001), emphasising the increased vulnerability of children growing up in FCAS countries (Khara et al., 2018).

**Table 4:** Studies that measured prevalence of concurrence at population level and within SAM treatment programmes

Study (first author/year)	Country	Population	Prevalence findings
**Population level data**			
Garenne (2018)	Senegal	Children 6–59 m	Wasting 16.3%
			Stunting 24.2%
		12,638 measures	Concurrence 6.2%
Harding et al. (2018a)	6 countries—South Asia	Children 0–59 m	Wasting 15.7%,
			Stunting 40.1%
		*n* = 62,509	Concurrence 6%
Harding et al. (2018b)	6 countries—South Asia	Children 0–59 m	Wasting 19.4%
			Stunting 38.35%
		*n* = 252,797	Concurrence 6.11
Khara (2017)	84 countries	Children 0–59 m	Wasting 8.8%
			Stunting 33.0%
		*n* = 570,930	Concurrence 3.0%
Kinyoki (2016)	Somalia	Children 0–59 m	Wasting 21%
			Stunting 31%
		*n* = 73,778	Concurrence 9%
Mutanga 2020	6 countries—South East Asia	Children 0–59 m	Wasting 8.9%
			Stunting (not individually presented)
		*n* = 47,481	Concurrence 1.65%
Odei Obeng‐Amoako, Karamagi, et al. (2020)	Uganda	Children 6–59 m	Stunting 33.58
			Wasting 12.03%
		*n* = 32,962	Concurrence 4.96%
Reese‐Masterson et al. (2016)	Kenya	Children 6–23 m	Wasting 8.8%
		227	Stunting 28%
			Concurrence 5%.
Saaka and Galaa (2016)	Ghana	Children 0–59 m	Wasting 4.7%
		*n* = 2720	Stunting 17.9%
			Concurrence 1.4%.
Sage (2017)	Guinea‐Bissau	Children 6–59 m	Wasting 6%
			Stunting 30%
		*n* = 6602	Concurrence 2.4%
Schoenbuchner et al. (2019)	Gambia	Children 0–23 m	Wasting 18% in boys/12% in girls^a^
		*n* = 3867	Stunting 39%^a^
		28,403 measures	Concurrence 9% in boys/5% in girls
Victoria, 2015	8 countries	Newborns	Wasting 3.4%
			Stunting 3.8%
		*n* = 60,206	Concurrence 0.7%
**SAM treatment data**			
Imam et al. (2020)	Nigeria	472 children in SAM treatment programme	Stunting 82.8%
Ngari (2018)	Kenya	1169 children admitted for SAM treatment	Stunting 69%
Odei Obeng‐Amoako, Myatt, et al. (2020)	Uganda	788 children in SAM treatment programme^b^	Stunting 48.7%

^a^ Peaks in wasting at 1 year and stunting at 24 months.

^b^ MUAC admission criteria in use to define wasting.

Population‐level data show that wasting, stunting and concurrent wasting and stunting are all more prevalent in boys than girls (Khara et al., 2018; Myatt et al., 2018; Odei Obeng‐Amoako, Karamagi, et al., 2020; Odei Obeng‐Amoako, Myatt, et al., 2020) and that wasting is higher in younger children while stunting is higher in older children. In the case of concurrent wasting and stunting, the peaks seem to appear between 12 and 30 months (Garenne et al., 2019; Imam et al., 2020; Khara et al., 2018; Mertens, Benjamin‐Chung, Colford, Coyle, et al., 2020; Myatt et al., 2019; Odei Obeng‐Amoako, Myatt, et al., 2020), with younger children and males being most affected (Odei Obeng‐Amoako, Wamani, et al., 2020). In Senegal, a change of direction was observed in risk with age whereby males were more likely to be concurrently wasted and stunted below the age of 30 months but less likely to be wasted above 30 months at the same level of stunting (Garenne et al., 2019).

SAM treatment programme data also indicates that concurrent wasting and stunting are more prevalent in boys and younger children (Imam et al., 2020;Isanaka et al., 2019; Odei Obeng‐Amoako, Wamani, et al., 2020). Data from an outpatient therapeutic programme (OTP) programme in Uganda showed that, despite higher overall admission in females, there were more males with concurrent wasting and stunting within the admitted group (Odei Obeng‐Amoako, Wamani, et al., 2020).

### 3.9 Mortality implications of concurrent wasting and stunting

We identified six studies that explored the mortality implications of concurrent wasting and stunting. Overall, studies show that children with concurrent wasting and stunting are at high risk of mortality (Garenne et al., 2019; McDonald et al., 2013; Mertens, Benjamin‐Chung, Colford, Coyle, et al., 2020; Myatt et al., 2018; Myatt et al., 2019; Pomati & Nandy, 2019). A meta‐analysis of 10 countries (McDonald et al., 2013) showed that children who are wasted, stunted and underweight had a 12‐fold elevated risk of mortality compared with those with no deficit. A later analysis of 51 countries demonstrated that all children who are wasted and stunted are also underweight and, therefore, the same elevated mortality estimate is applicable (Myatt et al., 2018). A recent longitudinal analysis of eight cohorts of children showed that all measures of early growth failure were significantly associated with a higher risk of death by age 24 months and those most strongly associated with death were children severely underweight before age 6 months, children with concurrent wasting and stunting and children under 6 months who were persistently wasted (Mertens, Benjamin‐Chung, Colford, Coyle, et al., 2020).

### 3.10 Wasting treatment outcomes and stunting

As stated above, stunting is highly prevalent among wasted children admitted to therapeutic feeding programmes (TFPs) (Isanaka et al., 2019; Odei Obeng‐Amoako, Wamani, et al., 2020), and there is some evidence of the influence of this on treatment response although, due to limited resources, length/height is not always measured upon admission to TFPs and therefore may be underreported. We identified six studies that assessed SAM treatment outcomes for children who are concurrently wasted and stunted with some inconsistencies in results. Data from Niger found the response to SAM treatment was independent of stunting with no difference in weight gain during or after treatment or in mean time to recovery (Isanaka et al., 2019). Conversely, data from Uganda found lower recovery rates in stunted children compared with nonstunted children during SAM treatment (58.0% vs. 65.4%; *p* < 0.037), higher rates of non‐response (18.7% vs. 9.8%; *p* < 0.001) but greater weight gain (2.2 g/kg/day vs. 1.7 g/kg/day; *p* = 0.004). MUAC gain did not differ between groups (Odei Obeng‐Amoako, Wamani, et al., 2020). Similarly, in Malawi, in a study examining children experiencing relapse after treatment for MAM, those who experienced a negative change in HAZ were more likely to experience relapse to MAM or SAM (OR = 1.72 ± 0.20, *p* < 0.001) (Stobaugh et al., 2018).

Given recent concerns that the provision of therapeutic foods might lead to excess fat accretion in stunted children contributing to future overweight, obesity and noncommunicable disease (Hawkes et al., 2020), we reviewed studies which assessed weight gain and body composition following nutritional therapy. The studies suggest that while stunting might affect response to treatment, there is no evidence of an effect of concurrent wasting and stunting on increased fat accumulation with the use of lipid based nutrient supplements (LNS) for either MAM or SAM (Binns & Myatt, 2018; Fabiansen et al., 2017, 2018; Kangas et al., 2020; Wells, Devakumar, et al., 2019).

Finally, we identified one study that explored longer term outcomes (1 to 7 years after treatment) for 378 children after SAM treatment including linear growth (Lelijveld et al., 2016). The data showed some recovery in the height of previously wasted children, but they still demonstrated more severe stunting than controls. Body composition assessment showed smaller calf and MUAC measurements suggesting reduced peripheral mass compared with control, and smaller hip circumference and larger or similar waist circumference suggesting an unhealthy ratio of core to gluteo‐femoral fat. In body composition assessment, cases also had lower FFM but similar fat mass compared with community controls after adjustment for age differences.

### 3.11 Anthropometric indices and the identification of risk

We found nine studies that assessed the use of different anthropometric indices and the implications of these on caseload and identifying the most vulnerable undernourished children. Many of these studies are rooted in the recent recognition that concurrent wasting and stunting lead to heightened mortality risk (Myatt et al., 2018) and prompt a re‐examination of risk and how best to identify it. Longitudinal analysis on data from Senegal found that the combined use of WAZ and MUAC identified all near‐term deaths associated with concurrent wasting and stunting and with severe wasting as defined by WHZ < −3 (Garenne et al., 2019). The lowest WAZ threshold that detected all deaths was <−2.8. Data from Niger similarly showed MUAC to be the best predictor of mortality in children 6–59 months followed by WAZ (O'Brien et al., 2020). Analysis of 16 cross‐sectional studies found stunting to be associated with WHZ < −2 and MUAC <125 mm but more strongly associated with MUAC <125 mm. The findings from these studies suggest WAZ identifies children with a high risk of mortality and that MUAC and WHZ might not (Odei Obeng‐Amoako, Myatt, et al., 2020); therefore, the use of MUAC with the addition of WAZ might effectively identify those children at most risk and in need of some level of treatment.

### 3.12 Ongoing research priorities on the relationship between wasting and stunting

We identified one study that focused on the identification of research priorities for concurrent wasting and stunting (Bhutta et al., 2016). This was a Child Health and Nutrition Research Initiative (CHNRI) exercise that identified top‐ranked research questions. In addition to this, we also found research recommendations in several studies. In particular, the need was highlighted to better understand the biological processes and causal pathways, for example, the role of gut health/inflammation, body composition and its relation with anthropometric indicators and functional outcomes, the contribution of lean and fat tissue during and after recovery from SAM (Briend, Khara et al., 2015) and the role of environmental factors and patterns of malnutrition (Angood, 2016).

Among the studies reviewed, prevention stood out as one of the key gap areas for further research that examines the interventions needed to halt and prevent the spiralling of vulnerabilities associated with early growth deficits. Studies called for research that focused on identifying interventions to improve maternal nutrition and prevent the risk of being born wasted or stunted or concurrently wasted and stunted, the magnitude of risk between birth and 3 months of age and interventions to mitigate seasonal peaks (Angood, 2016). Studies also highlighted the need for operational research to better understand the programmatic and cost implications of implementing WAZ and MUAC for targeting and caseload and to examine which treatment protocol approaches support the most vulnerable with the highest impact (Angood, 2016). In terms of treatment, some studies called for research to understand if longer treatment time or posttreatment interventions for SAM would allow for fuller recovery (Kangas et al., 2020) and the optimal RUTF formulation to promote linear growth during and after SAM treatment.

## 4 DISCUSSION

A significant and still‐growing body of evidence supports the existence of a strong relationship between wasting and stunting, which carries important implications for policy and practice. Wasting and stunting, driven by common factors, frequently occur in the same child, either at the same time or through their life course, with important interactions between them. This demonstrates the need for integrated policy and programme considerations and common prevention strategies.

One of the key findings from this review relates to the peak age of wasting and stunting. Research has previously explored the timing of growth faltering (Victora et al., 2010), but evidence reviewed here shows that the peak in incidence of both wasting and stunting is from birth to 3 months with implications for further deterioration in infancy and childhood. This finding offers new insights into how early experiences and underlying factors can lead to the accumulation of nutrition deficits and suggests that a greater focus on the youngest children and what will prevent their wasting and stunting is required. The increased risk of death by age 24 months illustrated following early growth faltering also points towards the need to place prevention of LBW and early growth failure high on the agenda for global health and nutrition stakeholders. To do this, it is widely recognised that innovative prevention programming that combines interventions targeting the health and nutrition status of women of reproductive age and pregnant women is needed (Bhutta et al., 2013; da Silva Lopes et al., 2017). Improvement in some of these early indicators (for example, birth length, maternal weight, birth order, maternal education levels, wealth indicators) has the potential to prevent 20–30% of the incidents of stunting and wasting (Mertens, Benjamin‐Chung, Colford, Coyle, et al., 2020).

This review underscores the finding that both wasting and stunting are interconnected processes linked to various deprivations in a child's environment and that of their mothers (both in‐utero and during infancy and childhood) and which lead to physiological and development stresses with consequences for body composition and physiological function. Although the evidence is growing and compelling, questions remain around the physiological mechanisms linking wasting and stunting and further research is warranted, particularly to better understand the critical junctures for halting the accumulation of vulnerabilities that are created as these processes interact. Some evidence suggests that, in addition to muscle and fat loss and hormonal imbalances, stunted children show deficits in the form of small organ size (Wells, Devakumar, et al., 2019) with potential deleterious implications for physiological function. Further research is needed to understand the full biological picture in order to intervene more effectively.

We have reviewed evidence that demonstrates that wasting can lead to stunting and, to a lesser extent, stunting can increase the risk of wasting. The former direction is supported by evidence of a mechanism whereby adequate weight and the absence and/or management of underlying morbidities is needed before linear growth can take place (Garenne, 2020; Isanaka et al., 2019; Ngari et al., 2019; Richard, Black, Gilman et al., 2012; Schoenbuchner et al., 2019). Previously published studies have shown similar findings that wasted children only demonstrate height growth once their weight for height is regained (Dewey et al., 2005) and where seasonal conditions are favourable (Maleta et al., 2003). These findings highlight the importance of the integrated medical and nutritional care of children receiving wasting treatment to ensure the effects of wasting on linear growth are minimised. While severe and/or repeated episodes of wasting may contribute to stunting, the higher prevalence of stunting cannot be solely explained by previous wasting. There are many drivers of stunting and many countries where stunting levels are high but wasting prevalence levels are low (GNR, 2020). The importance of wasting as a driver of stunting is therefore likely to vary by context. The evidence that shows stunting leading to wasting provides a new perspective on understanding how wasting and stunting are interrelated although the relationship is weaker. Further research to understand the mechanisms behind this would be informative for identifying programmatic implications.

Children identified as concurrently wasted and stunted have a dual burden of impact on body composition, which might explain the high risk of mortality associated with having both conditions. The cumulative increased risk of death from concurrent wasting and stunting undermines any rationale for different interventions addressing separate forms of undernutrition. Instead, treatment strategies need to shift focus to consider risk of death as paramount to targeting rather than specific categories of anthropometric cut‐offs to define wasting while working alongside prevention. Targeting interventions by season or by population subgroups defined by sex, socio‐economic status, maternal and child birth characteristics might help to focus preventive interventions to reduce the burden of postnatal growth failure (Mertens, Benjamin‐Chung, Colford, Coyle, et al., 2020).

Most of the prevalence studies in this review reported wasting, stunting and concurrent wasting and stunting as point prevalence. We have presented evidence that demonstrates problems in the underestimation of the actual burden of wasting as children can move in and out of periods of this acute condition throughout the year. Wells, Briend, et al. (2019) argue that the reliance on population‐level data describing stunting and wasting gives a profoundly misleading representation of the complexity of the causes of undernutrition and unnecessarily narrows programme and policy approaches to separate prevention and treatment of undernutrition rather than combined understanding and approaches. The design and implementation of nutrition programme and policy should therefore consider how incidence might inform more effective targeting of programme resources.

One of the secondary findings from the work identified in this review is higher prevalence of concurrent wasting and stunting in males. Although the concept of higher levels of male undernutrition is not new, the work here has renewed interest in understanding the reasons for these differences. A recent systematic review of sex differences in undernutrition showed that boys are more likely to be wasted, stunted and underweight compared with girls (Thurstans et al., 2020). There are some nuances in sex and age patterns whereby males appear to be more vulnerable in early years, but in some contexts, the risk is inversed as age increases, making girls more vulnerable (Garenne et al., 2019). This may be indicative of the varying influence of sociological factors over biological factors over time. Programme data collection, surveillance systems and national and local survey indicators should not only disaggregate all data by age and sex, but should also be modified to include the calculation of concurrent wasting and stunting (Odei Obeng‐Amoako, Myatt, et al., 2020).

The findings regarding response to SAM treatment for children who are both wasted and stunted are inconsistent. Overall, evidence suggests that outcomes are suboptimal for children with concurrent wasting and stunting. Where positive treatment outcomes are reported, this might be reflective of survivor bias. What is clear is that TFPs need to be optimised to identify most at‐risk children including those who are concurrently wasted and stunted (Bergeron & Castleman, 2012; Khara & Dolan, 2014). Likewise, the evidence presented above that wasted children often go on to experience further episodes of wasting (Schoenbuchner et al., 2019) and that wasting leads to stunting highlights the importance of strengthening the links between wasting and stunting prevention programmes. Children who have been enrolled in SAM treatment should be targeted to prevent further episodes. While few interventions have been shown to successfully treat stunting, what is not clear from this review is whether treatment of wasting could be adapted to better lay the foundation for linear growth (Briend, Khara, et al. 2015). For example, is the lack of gain in height solely related to the body's focus on weight gain, related to the RUTF formulations in use and lack of micronutrients to support bone growth or to the timeframe of the intervention? In the case of fat accumulation and overweight/obesity risk, findings from this review seem to allay concerns regarding the risk of excess fat accretion in stunted children.

This review highlights findings that support further operational research into the anthropometric identification and assessment of undernutrition. Evidence shows that MUAC and WAZ are the best measures to identify mortality risk (Myatt et al., 2019) including in infants under 6 months of age (Mwangome et al., 2017). The association between young age and low muscle mass also highlights young infants, in particular those born small for gestational age (SGA), as a priority group (Briend, Khara et al., 2015). This age group is often excluded from programming due to the complexities of the identification of undernutrition. In a recent study, the use of MUAC and WAZ were found to identify high‐risk infants under 6 months. LBW, MUAC <9 cm and WAZ ‐ < 3 *Z* score at birth were each positively associated with increased risk of mortality during the first year of life (Mwangome et al., 2019). This has important implications in reaching the most vulnerable children in a way that is programmatically practical and potentially less open to measurement error than WHZ.

The findings in this review on the peak timing of wasting and stunting suggest that policy and practice need to address undernutrition in a way that considers the life cycle of undernutrition, from preventative interventions targeted to women of reproductive age through to treatment when undernutrition occurs in the young child. A significant degree of child undernutrition is established before birth (Christian et al., 2013), indicating the need for greater coordination between interventions targeting adolescent girls and mothers and those aiming to prevent child undernutrition (Wells, Briend, et al., 2019). The evidence on the relationship between stunting and wasting suggests that the divide in tackling undernutrition needs to be addressed at all levels including financing arrangements that should promote longer term funding, particularly in protracted crisis contexts, to allow for investments in prevention as well as more immediate life‐saving interventions (MQSUN+, 2020).

The strength of the evidence has come a long way since the original review in 2014, and continued robust research on the priorities laid out above will be key to furthering our understanding of the relationship between wasting and stunting. This would serve to better prioritise prevention and treatment‐focused interventions in all contexts where undernutrition is a concern.

The strength of this review lies in the systematic approach taken, but we recognise some limitations. Our search strategy might have introduced some bias in the literature that we included. The findings have demonstrated the overlap in wasting and stunting and the utility of measures of underweight in capturing this. As we did not include the term ‘underweight’ in our search, there is a chance that we missed relevant literature pertaining to underweight only. However, papers would only have been included if they also mentioned wasting and stunting and so should have been identified by the search. We also do not feel that the overall message that a child should be viewed in a more holistic way would be changed. Similarly, our findings demonstrate that, in many instances, children are born wasted and/or stunted and therefore LBW. While we did not include the terms ‘low‐birthweight’ or ‘preterm’ in our search, we have presented research that highlights the need for prevention through maternal and newborn interventions. Finally, our search may have had reduced sensitivity with the limits we applied related to relationship and association. However, we felt this was necessary to manage the large quantity of literature related to both wasting and stunting given our interest in the relationship between the two.

For all of the above limitations, we feel our request to members of the WaSt TIG SWG to highlight relevant additional literature has contributed to minimising the effects given their expertise and ongoing work in the field of maternal and child health and nutrition. We recognise the limitations of cross‐sectional data throughout the text, and this is particularly relevant for assessing causal associations and incidence. In terms of associations, all cross‐sectional evidence that we have presented is supported by longitudinal data, providing robust support to the findings. Finally, there might also be a risk of survivor bias in included studies, particularly those related to long‐term outcomes of wasting and stunting.

## 5 CONCLUSION

The ongoing accumulation of evidence since the 2014 review demonstrates progress in improving the understanding and awareness of the relationship between wasting and stunting. The findings of this review are supportive of a strong relationship between these two manifestations of undernutrition and provide a better understanding of which groups should be considered at risk and therefore prioritised for treatment.

Evidence on the cumulative effects of nutritional deficits, and therefore risk over the life course of a child beginning in‐utero, demonstrates the need for a more integrated approach to prevention and treatment strategies in order to interrupt this process. To achieve this, further progress is needed to overcome the divide that has typified undernutrition policy, programme, financing and research initiatives.

## CONFLICTS OF INTEREST

The authors declare that we have no conflict of interest.

## CONTRIBUTIONS

ST led in the development of the study protocol which was reviewed by TK and CD. ST conducted the search with a second review by NS. ST led in the analysis and the write up of the manuscript with regular contributions from all authors. All authors have read and approved the final manuscript.

## Data Availability

Data sharing is not applicable to this article as no datasets were generated or analysed during the current study.
